# Acaricidal and synergistic activity of essential oils, their binary combinations, and nanoemulsions against larvae and unfed adult stages of brown dog tick *Rhipicephalus sanguineus* (Acari: Ixodidae)

**DOI:** 10.1186/s12917-025-05214-9

**Published:** 2026-01-20

**Authors:** Hoda S.M. Abdel-Ghany, Fathalla Ayoob, Bassma S.M. Elsawy, Heba F. Alzan, Abdelghany A. Youssef, Sobhy Abdel-Shafy

**Affiliations:** 1 Department of Parasitology and Animal Diseases, Veterinary Research Institute, National Research Centre, Dokki, Giza Egypt; 2 Ticks and Tick-Borne Diseases Research Unit, Veterinary Research Institute, National Research Centre, Dokki, Giza, 12622 Egypt; 3 Department of Tanning Materials and Leather Technology, Chemical Industries Research Institute, National Research Centre, Dokki, Giza Egypt; 4 Medicinal and Aromatic Plants Research Department, Pharmaceutical Industries Research Institute, National Research Centre, Dokki, Giza Egypt

**Keywords:** Adulticidal activity, Essential oils, Larvicidal, Nanoemulsion, Rhipicephalus sanguineus, Synergism

## Abstract

The growing resistance of ticks to acaricides has made tick management a global concern. Various studies now focus on identifying natural products with acaricidal properties that are environmentally safe. The current research investigates the effect of clove (*Syzygium aromaticum*), mint (*Mentha longifolia*), and geranium (*Pelargonium graveolens*) essential oils (EOs), along with their combinations and nanoemulsion forms (NE), against *Rhipicephalus sanguineus* (*R. sanguineus*) larvae and unfed adults. The chemical composition analysis using Gas chromatography-mass spectrometry (GC-MS) identified eugenol (68.02%) and caryophyllene (20.19%) in clove EO, pulegone (33.48%) and l-menthone (22.28%), in mint EO, and citronellol (22.24%), γ-Eudesmol (13.2%), and Geraniol (10.35%) in geranium EO as the main active constituents. Nanoemulsions were characterized using Transmission electron microscopy (TEM) and dynamic light scattering (DLS) to assure the mean particle size and the mean particle distribution. The reported particle sizes show noticeable differences (e.g., TEM ranges from 47.8 to 173 nm vs. DLS ranges from 80 to 244.2 nm), and the PDI values are relatively high (e.g., 0.55 for geranium NE). According to LC_50_ values, the most effective materials against larvae were mint NE (LC_50_ = 0.36%), followed by the (clove + mint) EO combination (LC_50_ = 1.43%), and then clove EO (LC_50_ = 1.68%). While the most effective materials against unfed adults were clove NE (LC_50_ = 1.63%), followed by (mint + geranium) combination (LC_50_ = 4.93%), and then geranium EO (6.1%). The EOs combinations exhibited synergistic efficacy against larvae and unfed adults, with the synergistic factor (SF) > 1. The (clove + mint) binary combination showed a strong synergistic effect against larvae, with SF of 2.46. Furthermore, the (mint + geranium) binary combination exhibited the highest synergistic activity against unfed adults with SF (1.39). These findings suggest that EOs, especially in NE form, represent promising alternatives to chemical acaricides. Future work will focus on studying the possibility of combining NE and evaluating its effect against ticks. More importantly, performing safety evaluation and in vivo study of these formulations to develop efficient and environmentally friendly tick-control materials.

## Introduction

Ticks are obligate hematophagous ectoparasites found worldwide and infest various vertebrate hosts, including humans and animals [1, 2]. The brown dog tick *Rhipicephalus sanguineus* (*R. sanguineus*) *sensu lato* (*s.l*.) is a widely distributed ixodid tick that prefers dogs as a host [3]. Heavy infestations of this tick can cause anemia, skin lesions, inflammation, and allergic reactions. *Rhipicephalus sanguineus* also transmits diverse pathogens as *Babesia vogeli*, *Ehrlichia canis*, *Anaplasma platys* and *Hepatozoon* canis [4]. Moreover, it can parasitize humans, posing public health risks [5]. So, controlling this tick species is thus crucial for both medical and veterinary purposes. The most prevalent technique for ectoparasite control is the application of chemical acaricides. Although several chemical-based products are available to control *R. sanguineus*, dog owners and veterinarians have stated that certain chemical acaricides have become ineffective, potentially due to the development of tick resistance [6, 7]. Thus, new tick management protocols have been investigated to develop products harmless to vertebrates, but efficient in tick control. Numerous studies have demonstrated that several plant-derived materials harm ticks, so essential oils represent potential alternatives to acaricides [8, 9]. Among these, clove EO (*Syzygium aromaticum*, family Myrtaceae) has been shown to have numerous acaricidal activities against ticks such as *Rhipicephalus microplus* [10–12] and it also has potent ovicidal and adulticidal effects against *Hyalomma scupense* [13]. In addition to its acaricidal efficacy, clove EO has antioxidants, antibacterial, and antifungal activity [11]. Furthermore, mint (*Mentha longifolia*, family Lamiaceae), commonly known as horse mint or wild mint, is used to repel insects, skin infections, inflammation, scabies, and insect bites [14, 15]. The mint EO has been reported to exhibit acaricidal activity against *Rhipicephalus turanicus* tick [16]. Moreover, it affects *Culex quinquefasciatus*, *Aedes aegypti*, and *Anopheles stephensi* [17]. Similarly, geranium (*Pelargonium graveolens;* family Geraniaceae.) is an aromatic medicinal herb utilized in perfume, insect repellent, and as a flavoring agent [18]. Its EO affects the reproductive performance of cattle ticks *R. microplus* females [19], in addition to its acaricidal efficacy against *R. annulatus* [20] and repellent activity against *Ixodes ricinus* [21]. Although several studies have documented the acaricidal properties of clove, mint, and geranium EOs against various tick species, including *R. microplus*, *H. scupense*, and *I. ricinus*, information about their effects on *R. sanguineus* remains limited. Each essential oil possesses a unique chemical composition and distinct mechanism of action, which may target different physiological pathways in arthropods. A binary combination of two or more compounds can create novel botanical pesticides containing substances that have synergistic effects. This synergism increases mortality rates and offers environmental, toxicological, and production advantages by needing lower quantities of each compound [22, 23]. Moreover, combining two or more substances with diverse modes of action can delay resistance development [24]. For example, the combination of geranium, oregano, and thymol enhanced the acaricidal activity against *R. sanguineus* ticks [25]. Likewise, clove and lemon grass EO combinations exhibited enhanced acaricidal activity against *R. microplus* larvae compared to individual oil treatments [12]. Moreover, thymol and eugenol mixtures showed a synergistic effect, enhancing their acaricidal activity against *R. sanguineus* ticks [26]. However, the practical application of essential oils is often limited by their volatility and poor solubility. Nanoemulsion (NEs) formulation is another method used to increase the efficacy of EOs because of their stability and reduced droplet size, which enhances biological activity [27]. Several NE formulations proved their potency against *R. sanguineus* ticks as myrrh, patchouli, and cypress NE [28]; *Thymus vulgaris* NE [29], and d-limonene NE [30]. Geranium NE proved its potent effect against different developmental stages of *R*. annulatus ticks [20]. While the individual acaricidal effects of clove, mint, and geranium EOs have been studied separately on various ticks, their binary combinations against *R. sanguineus* remain unexplored. Furthermore, despite the promise of nanoemulsions, the efficacy of nanoformulations of mint and clove oil against ticks is largely unknown. Therefore, this study aimed to evaluate the acaricide activity of the clove, mint, and geranium EOs along with their combinations and nanoemulsion forms against *R. sanguineus* larvae and adults.

## Materials and methods

### *Rhipicephalus sanguineus *tick

**Fig. 1:**
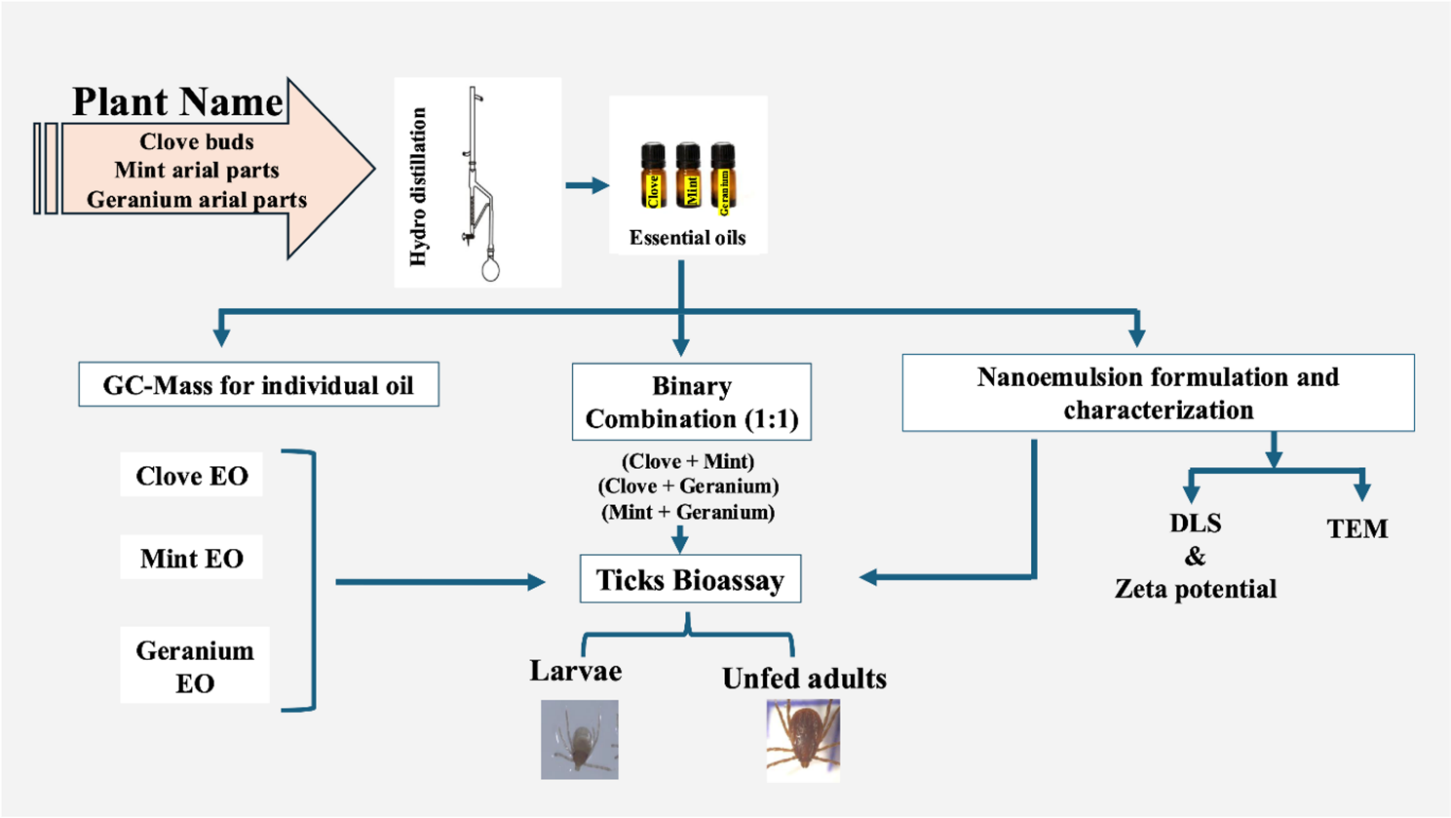
Schematic overview of the experimental workflow

The experimental design is represented in Fig. 1. Larvae and unfed adults were obtained from an established *Rhipicephalus sanguineus* colony at the Parasitology and Animal Diseases Department, Veterinary Research Institute, National Research Centre, Dokki, Giza, Egypt (30.0444° N, 31.2357° E). Fully engorged females were incubated at 25 °C ± 1 °C and 75%–80% relative humidity (RH) for egg laying and hatching of larvae. Some hatched larvae were utilized in the larval immersion test, and others were fed on healthy rabbits to obtain fully engorged larvae. The engorged larvae were incubated until they molted into unfed nymphs, which then fed on rabbits to become fully engorged nymphs [31]. The collected nymphs were incubated until they molted into unfed adults for use in the adult immersion test. All tick stages were fed using the capsule technique [32].

### Plant materials

*Syzygium aromaticum* (clove), *Mentha longifolia* (mint), and *Pelargonium graveolens* (geranium) were obtained from the Institute of Medicinal and Aromatic Plants, Egyptian Ministry of Agriculture, Cairo, Egypt.

### Essential oil extraction

Essential oil from clove buds and the aerial portions of mint and geranium were extracted for 3 h using the hydro-distillation method (Clevenger apparatus) according to Clevenger [33]. The condensed volatile oil was collected in glass bottles. The obtained volatile oil was dried over anhydrous sodium sulphate. The obtained oil was stored at 4 °C in brown vials until analysis.

### Gas chromatography-mass spectrometry (GC-MS) analysis

Analysis of the oils was conducted using gas chromatography (Agilent 8890 GC System), coupled to a mass spectrometer (Agilent 5977B GC/MSD) and equipped with a HP-5MS fused silica capillary column (30 m, 0.25 mm i.d., 0.25 mm film thickness). The oven temperature was maintained initially at 50 °C, then programmed from 50 to 220 °C at a rate of 5 °C/min and from 220 °C to 280 °C at a rate of 15 °C/min, then held for 7 min at 280 °C. Helium was used as the carrier gas at a flow rate of 1.1 mL/min. The essential oil was dissolved in diethyl ether (30 µL essential oil/mL diethyl ether), and then 1 µL of this solution was injected into the GC with a split ratio of 1:50. The temperature of injection was 230 °C. Mass spectra in the electron impact mode (EI) were obtained at 70 eV and scan m/z range from 39 to 500 amu. The isolated peaks were identified by matching them with data from the library of mass spectra (National Institute of Standard and Technology, NIST).

### Formulation of nanoemulsion containing essential oils

The nanoemulsion was prepared following the general formulation approach of Sugumar et al. [34], with fundamental modifications: the coarse emulsion was homogenized by magnetic stirring at 2000 rpm overnight without subsequent ultrasonication. Briefly, the oil-in-water nanoemulsion was formulated using an essential oil of clove, mint, and geranium, a non-ionic surfactant (tween 80), and water. The concentration of each essential oil was fixed for all the formulations to the surfactant at a ratio of 2:1. Tween 80 was purchased from Loba Chem, India. All chemicals and solvents were HPLC grade. Initially, the coarse emulsion was prepared by adding essential oil at a slow rate of one ml per minute to the organic phase containing water and surfactant (5%v/v) using a magnetic stirrer at 2000 rpm, and kept stirring overnight at room temperature. The physical stability of the nanoemulsion was monitored by visual inspection for any changes in color or phase separation at two-week intervals. No alteration in color or phase separation was observed, indicating that the formulation remained physically stable for at least three months after preparation [35].

### Characterization of nanoemulsion containing essential oils

#### Transmission electron microscope (TEM)

The nano-size images of the prepared clove, mint, and geranium NE samples were recorded by a high-resolution transmission electron microscope (HRTEM), JEOL model JEM-2100, which was used to study the particle shape and size of the prepared samples. Drops of the diluted preparations were deposited on a carbon-coated copper grid and then left to dry at room temperature for 10 min before investigation.

#### Particle size distribution analysis

The particle size distribution analysis was carried out with a Malvern Zetasizer 3000 HAS using a dynamic light scattering (DLS) technique at run time: 2 min, temperature: 23 °C, solvent: water, concentration: 1 mg/mL.

### Bioassay of the tested formulations against larvae and adult ticks

#### Larval immersion test (LIT)

The larval immersion test was based on the method described by Abdel-Ghany et al. [36] with some modifications in the time and the volume of the product used in the immersion of larvae. The three EOs, their binary combinations (at a ratio of 1:1), and the nanoemulsion were tested against larvae. In LIT, approximately 100 *R. sanguineus* larvae for each replicate were immersed in 1 ml of the tested formulation for 2 min. Five different concentrations were used, with three replicates for each one. The concentrations of clove, mint, and geranium EO, as well as their binary combinations (in a 1:1 ratio), were 10, 5, 2.5, 1.25, and 0.625%, prepared in 70% ethyl alcohol, while the concentrations of nanoemulsion for clove and mint were 3.75, 1.87, 0.93, 0.46, and 0.23% prepared in distilled water. For geranium, the concentrations of nanoemulsion were 15, 7.5, 3.75, 1.87, and 0.93%. The negative control was 70% ethyl alcohol, that used as a solvent for oils and distilled water for the NEs. The reference acaricide Sebacil^®^ (phoxim 50%, Bayer company, Germany) at a recommended dose of 1 ml/L was used as the positive control. The concentrations used in this study were based on a pilot study. The treated larvae were incubated at 25 °C ± 1 °C with 75% to 80% relative humidity (RH) and were examined after 24 h to detect the mortality rate. Larvae that could not move when gently stimulated by a fine brush were considered dead.

#### Adult immersion test (AIT) - Unfed adults

In AIT, 10-day-old unfed adults were immersed in 2 ml of the tested formulation for 2 min as outlined by Abdel-Ghany et al. [37]. Five different concentrations were tested, each with three replicates, and each replicate consisted of 10 unfed adult ticks (5 males and 5 females). The concentrations of clove, mint, and geranium oils, along with their binary combinations, were 20, 10, 5, 2.5, and 1.25%, while the concentrations of nanoemulsions were 15, 7.5, 3.75, 1.87, and 0.93%. The solvent control consisted of 70% ethyl alcohol for the oils and distilled water for the NEs. The reference acaricide Sebacil^®^ (phoxim 50%, Bayer Company, Germany) at a recommended dose of 1 ml/L was used as the positive control. The treated adult ticks were incubated at 25 °C ± 1 °C and 75%–80% RH and were examined over three successive days for the EOs and their binary combinations, and over seven successive days for the NEs treatment, as the essential oils and their binary combinations produced rapid effects due to their high volatility, while nanoemulsions exhibited slower release and delayed action but sustained effect on tick mortality. Mortality of unfed adults was assessed by naked eye and under a stereomicroscope. Ticks that showed no movement when gently stimulated with a fine brush and failed to respond were considered dead.

### Statistical analysis

The percent mortality of *R. sanguineus* larvae and unfed adults exposed to EOs, their combinations, and NEs were statistically analyzed using one-way ANOVA tests with F-tests, followed by the Tukey test, employing the SPSS program version 20. Mortalities for larvae were corrected with Abbott’s formula [38]. The lethal concentration (LC_50_) values for larvae and unfed adults were determined through regression equation analysis of the probit-transformed mortality data. The dose-response data were analyzed using the probit method, utilizing Ehab software [39]. To evaluate the synergistic or antagonistic activity between essential oil binary combinations, the synergistic factor (SF) was calculated according to Suwannayod et al. [40] as follows:

$$\:\mathrm{S}\mathrm{F}\:=\frac{\:\:{\mathrm{L}\mathrm{C}}_{50}\:\mathrm{o}\mathrm{f}\:\mathrm{i}\mathrm{n}\mathrm{d}\mathrm{i}\mathrm{v}\mathrm{i}\mathrm{d}\mathrm{u}\mathrm{a}\mathrm{l}\:\mathrm{o}\mathrm{i}\mathrm{l}\:\:}{{\mathrm{L}\mathrm{C}}_{50}\:\mathrm{o}\mathrm{f}\:\mathrm{c}\mathrm{o}\mathrm{m}\mathrm{b}\mathrm{i}\mathrm{n}\mathrm{a}\mathrm{t}\mathrm{i}\mathrm{o}\mathrm{n}}$$

SF > 1 indicates synergism; SF < 1 indicates antagonism.

## Results

### Active ingredients of essential oils by GC-MS analysis

The GC-MS analysis of the clove EO is displayed in Table 1. The output of the clove EO yield was 7.8%. Eugenol (68.02%) and Caryophyllene (20.19%) were the most abundant constituents. Considering mint EO, the production of essential oil from mint was 0.87%. The main compounds were Pulegone (33.48%), l-Menthone (22.28%), and Eucalyptol (12.03%) (Table 2). The yield of the geranium EO was 0.55%. The sample contained exactly 33 chemical compounds (Table 3). Citronellol (22.24%), γ-Eudesmol (13.2), Geraniol (10.35%), citronellyl formate (8.46%), and iso-menthone (5.65%) were the most abundant components.

**Table 1:** Chemical composition of essential oil extracted from clove flower buds by hydro-distillation

Peak	RT	RI_Cal_	RI_Lit_.	Oil constituent	Relative %
1	13.368	1197	1190	Methyl salicylate	0.46
2	18.035	1371	1366	Eugenol	68.02
3	18.306	1382	1374	Copaene < α- >	0.79
4	19.49	1428	1419	β-Caryophyllene	20.19
5	20.287	1460	1452	α-Humulene	3.81
6	21.5	1510	1505	α-Farnesene	0.74
7	21.939	1528	1522	δ-Cadinene	0.76
8	22.014	1531	1536	Eugenol acetate	5.24
EO Yield					7.8%

*RT* Retention time, *RI*_*Cal*_  Retention Index calculated using *n-alkanes* series (8:20), *RI*_*Lit*_  Retention index from literature

**Table 2:** Chemical composition of essential oil extracted from mint aerial parts by hydro-distillation

Peak	RT	RI_Cal_	RI_Lit_.	Oil constituent	Relative %
1	6.316	934	939	α-Pinene	1.68
2	6.68	948	954	Camphene	0.92
3	7.287	973	975	Sabinene	1.57
4	7.379	977	979	β-Pinene	2.44
5	7.697	990	990	β-Myrcene	1.09
6	8.731	1030	1029	D-Limonene	2.16
7	8.846	1034	1031	Eucalyptol	12.03
8	12.317	1160	1152	l-Menthone	22.28
9	12.554	1168	1162	iso-Menthone	2.18
10	12.612	1170	1169	Borneol	4.85
11	12.866	1179	1177	Isopulegone	1.98
12	13.29	1195	1188	α-Terpineol	3.09
13	13.807	1213	1205	Berbenone	0.55
14	14.772	1249	1237	Pulegone	33.48
15	15.06	1279	1252	Piperitone	1.18
16	17.394	1347	1343	Piperitenone	2.93
17	19.415	1425	1419	β-Caryophyllene	2.27
18	21.737	1519	1513	γ-Cadinene	0.51
19	24.717	1648	1640	tau.-Cadinol	2.83
EO yield					0.87%

*RT* Retention time, *RI*_*Cal*_ : Retention Index calculated using *n-alkanes* series (8:20), *RI*_*Lit*_ Retention index from literature

**Table 3:** Chemical composition of essential oil extracted from geranium aerial parts by hydro-distillation

Peak	RT	RI_Cal_	RI_Lit_.	Oil constituent	Relative %
1	10.677	1101	1096	Linalool	1.74
2	10.994	1112	1108	cis-Rose oxide	0.85
3	12.542	1168	1162	iso-Menthone	5.65
4	14.333	1233	1225	Citronellol	22.24
5	14.645	1244	1238	Neral	0.8
6	15.037	1258	1252	Geraniol	10.35
7	15.442	1273	1267	Geranial	0.68
8	15.551	1277	1273	citronellyl formate	8.46
9	16.262	1304	1298	Geranyl formate	2.35
10	17.59	1354	1348	α-Cubebene	0.76
11	18.266	1380	1376	α-copaene	0.56
12	18.52	1390	1384	(-)-β-Bourbonene	0.97
13	19.415	1425	1419	β-Caryophyllene	1.53
14	19.883	1444	1446	Citronellyl propionate	0.96
15	19.975	1448	1450	*cis*-Muurola-3,5-diene	0.74
16	20.131	1454	1454	**α-** Humulene	1.02
17	20.668	1475	1473	Geranyl propionate	0.99
18	20.951	1487	1482	Germacrene D	4.13
19	21.327	1502	1500	Bicyclogermacrene	0.73
20	21.95	1529	1523	**δ-** -Cadinene	3.24
21	22.551	1554	1550	α-Agarofuran	0.56
22	22.736	1562	1563	Geranyl butyrate	1.13
23	23.407	1590	1585	2-Phenylethyl tiglate	3.02
24	24.157	1623	1619	Cubenol < 1,10-di-epi->	0.73
25	24.307	1630	1632	γ-Eudesmol	13.2
26	24.44	1636	1634	Selina-1,3,7(11)-trien-8-one	0.65
27	24.526	1640	1637	epi- γ-Eudesmol	0.69
28	24.624	1644	1640	Agarospirol	1.03
29	24.763	1650	1649	tau-Cadinol	0.89
30	24.827	1653	1650	Eudesmol < β ->	1.43
31	24.985	1660	1653	Eudesmol < α->	4.29
32	25.167	1668	1668	Citronellyl tiglate	1.44
33	25.958	1702	1696	Geranyl angelate	2.18
EO yield					0.55%

*RT* Retention time, *RI*_*Cal*_ Retention Index calculated using *n-alkanes* series (8:20), *RI*_*Lit*_ Retention index from literature

The chemical classes of the extracted essential oils of clove, mint, and geranium are shown in Table 4. Eugenol and Eugenol acetate were the most abundant phenylpropanoid compounds found in clove bud oil (73.26). Sesquiterpene hydrocarbons, including β-Caryophyllene (20.19%) and α-Humulene (3.81%), accounted for 26%, whereas others, aromatic ester (methyl salicylate) classes made up 0.46%. In contrast, no oxygenated monoterpenes, sesquiterpenes, or monoterpene hydrocarbons were identified. Mint EO included volatile components that belonged to all terpene classes. Oxygenated monoterpenes made up 84.55%, followed by monoterpene hydrocarbons (9.86%), oxygenated sesquiterpenes (2.83%), and sesquiterpene hydrocarbons (2.78%). Finally, geranium EO was classified as oxygenated monoterpenes (42.3%), oxygenated sesquiterpenes (27.09%), sesquiterpene hydrocarbons (13.7%), and other substances such as esters (16.91%).

**Table 4:** Chemical classes of clove, mint and geranium essential oil extracted by hydro distillation

Chemical classes	Clove	Mint	Geranium
Monoterpene Hydrocarbons	0	9.86	0
Oxygenated Monoterpenes	0	84.55	42.3
Sesquiterpene Hydrocarbons	26.29	2.78	13.7
Oxygenated Sesquiterpenes	0	2.83	27.09
Phenylpropanoid compounds	73.26	0	0
Others	5.7	0	16.91

### Characterization of the prepared nanoemulsions

**Fig. 2:**
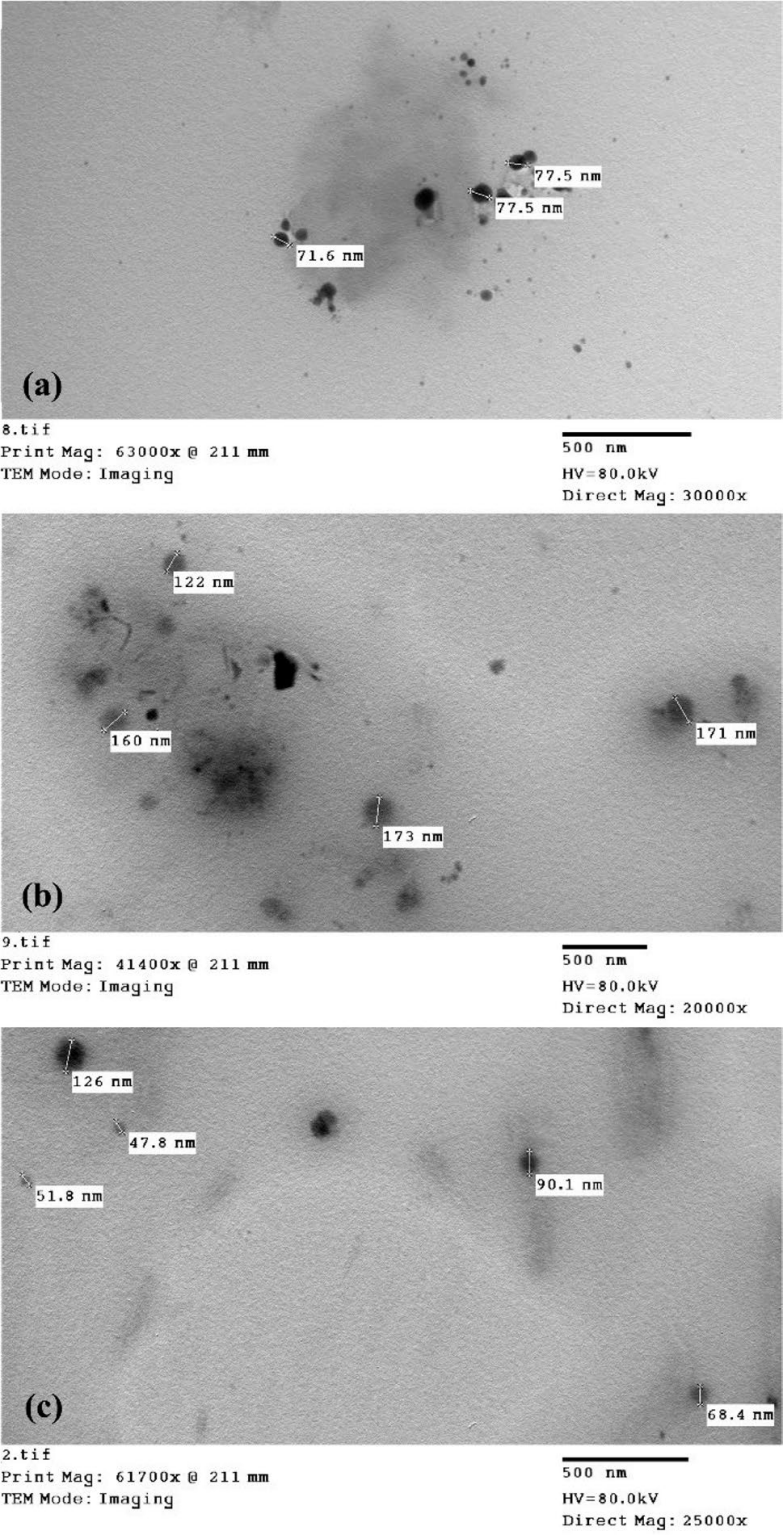
TEM images of essential oil nanoemulsions: (**a**) clove, (**b**) mint, (**c**) geranium

The prepared nanoemulsions showed a homogeneous, milky appearance. The morphology of the nanoemulsion (NE) containing 2% v/v essential oil as a fixed ratio for the three oils was screened by TEM. It was recorded that the droplets of NE were almost spherical, and the average particle size from TEM images ranges from 47.8 to 173 nm, which indicates successful preparation of nanomaterials (Fig. 2).

**Fig. 3:**
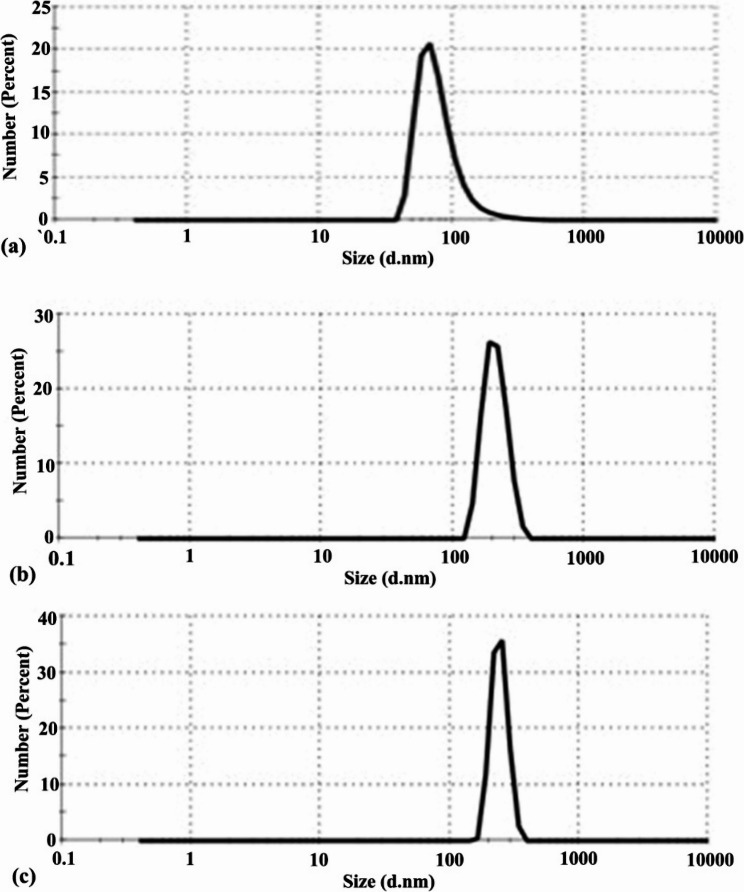
Particle size analysis of essential oil nanoemulsions: (**a**) clove, (**b**) mint, (**c**) geranium

**Fig. 4:**
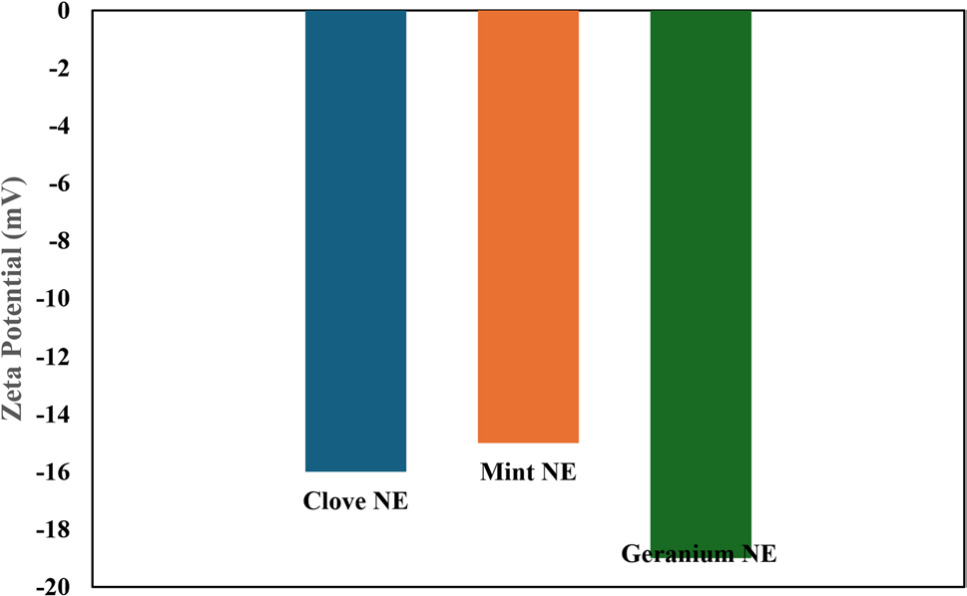
Zeta potential of clove, mint, and geranium essential oil nanoemulsions NE: nanoemulsion

Dynamic light scattering results (Fig. 3) show the average size of the particles distributed in the nano-emulsion (NE). The particle size and polydispersity index (PDI) were (80 nm & 0.286), (213.2 nm &0.331), and (244.2 nm & 0.55) for clove, mint, and geranium NEs, respectively. The zeta potential values demonstrated the formulations’ stability and surface charge properties. The nanoemulsion formulations showed negative zeta potential values of − 16 ± 3.34 mV, − 15 ± 2.24 mV, and − 19 ± 2.34 mV for clove, mint, and geranium, respectively, indicating stabilization through electric charge and steric hindrance provided by Tween 80 Fig. 4.

### Larvicidal effect of essential oils, their binary combinations, and nanoemulsions against *Rhipicephalus sanguineus*

**Fig. 5:**
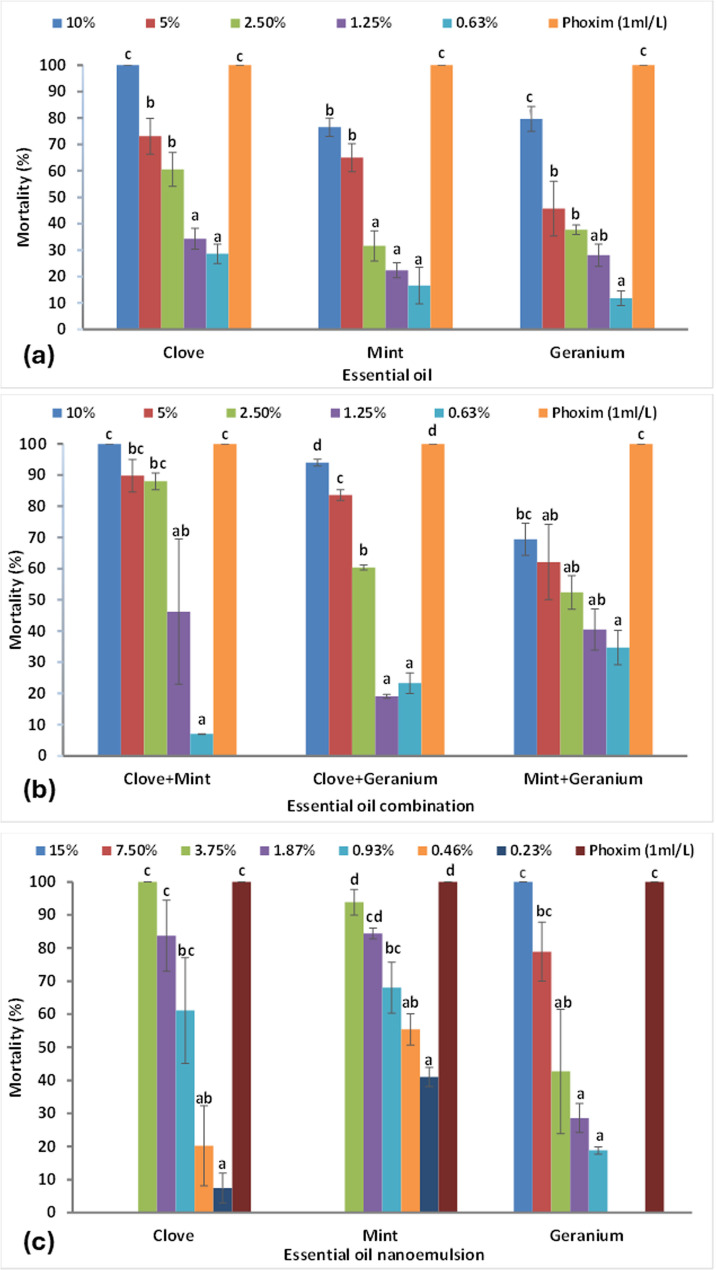
Corrected mortality of *Rhipicephalus sanguineus* larvae exposed to different formulations for 24 h: **(a)** clove, mint and geranium essential oils, **(b)** essential oil binary combinations (clove + mint), (clove + Geranium), (mint + Geranium), **(c)** clove, mint and geranium nanoemulsions

In LIT, the three essential oils had concentration-dependent effects as shown in Fig. 5a. Clove EO demonstrated the highest larvicidal efficacy with the lowest LC_50_ value (1.68%) compared with mint and geranium, which had LC_50_ values of 3.52% and 3.91%, respectively, after 24 h (Table 5). The reference acaricide (phoxim 1 ml/L) recorded 100% mortality. The larvicidal efficacy of the binary combinations varied depending on the oils involved, as presented in Fig. 5b. The LC_50_ values of the oil combinations were lower than those of the individual oils. The highest larvicidal activity against *R. sanguineus* larvae was observed in the (clove + mint) combination, with LC_50_ value of 1.43%, followed by (clove + geranium) at 1.95% and (mint + geranium) at 2.17% after 24 h (Table 5). All EO combinations except (mint + geranium), have a non-significant difference with phoxim (Fig. 5b). Essential oil combinations exhibited synergistic efficacy, with the synergistic factor exceeding 1, except for the slight antagonistic effect observed in the (clove + geranium) combination, which had a synergistic factor of 0.86. The (clove + mint) combination showed a strong synergistic effect against larvae, with a synergistic factor of 2.46 (Table 5).

**Table 5:** Lethal concentration of 50% of *Rhipicephalus sanguineus* larvae (LC_50_) treated with essential oils, their binary combinations, and nanoemulsions after 24 h, and synergistic factors of essential oils

Treatment	LC_50_ (%)	Confidence limits (%)	Slope ± SE	SF (Clove)	SF (Mint)	SF (Geranium)
Clove essential oil	1.68	0.61–3.11	1.86 ± 0.16	-	-	-
Mint essential oil	3.52	2.94–4.29	1.53 ± 0.15	-	-	-
Geranium essential oil	3.91	3.91–2.41	1.46 ± 0.15	-	-	-
Clove + Mint (1:1)	1.43	0.76–2.35	3.24 ± 0.25	1.17	2.46	-
Clove + Geranium (1:1)	1.95	0.94–3.5	2.11 ± 0.17	0.86	-	2.00
Mint + Geranium (1:1)	2.17	1.49–3.04	0.77 ± 0.13	-	1.62	1.8
Clove nanoemulsion	0.791	0.70–0.88	3.04 ± 0.21	-	-	-
Mint nanoemulsion	0.363	0.27–0.45	1.40 ± 0.15	-	-	-
Geranium nanoemulsion	3.175	1.53–5.93	2.24 ± 0.17	-	-	-

*SF* Synergistic factor, *SF > 1* Synergism, *SF < 1* antagonism

Figure 5c shows the larvicidal activity of the three nanoemulsions against *R. sanguineus* larvae. Among the three NEs, clove and mint NEs displayed the highest efficacy, with lower LC_50_ values of 0.79% and 0.36%, respectively. In contrast, the LC_50_ value of geranium was 3.17% after 24 h (Table 5). All NEs have a non-significant difference with phoxim (Fig. 5c). According to the comparison of LC_50_ obtained by the in vitro LIT, the mint NE (LC_50_ = 0.36%) was the most effective, followed by (clove + mint) EO combination (LC_50_ = 1.43%) and then the clove EO (LC_50_ = 1.68%).

### Adulticidal activity of essential oils, binary combinations, and nanoemulsions against *Rhipicephalus sanguineus*

**Fig. 6:**
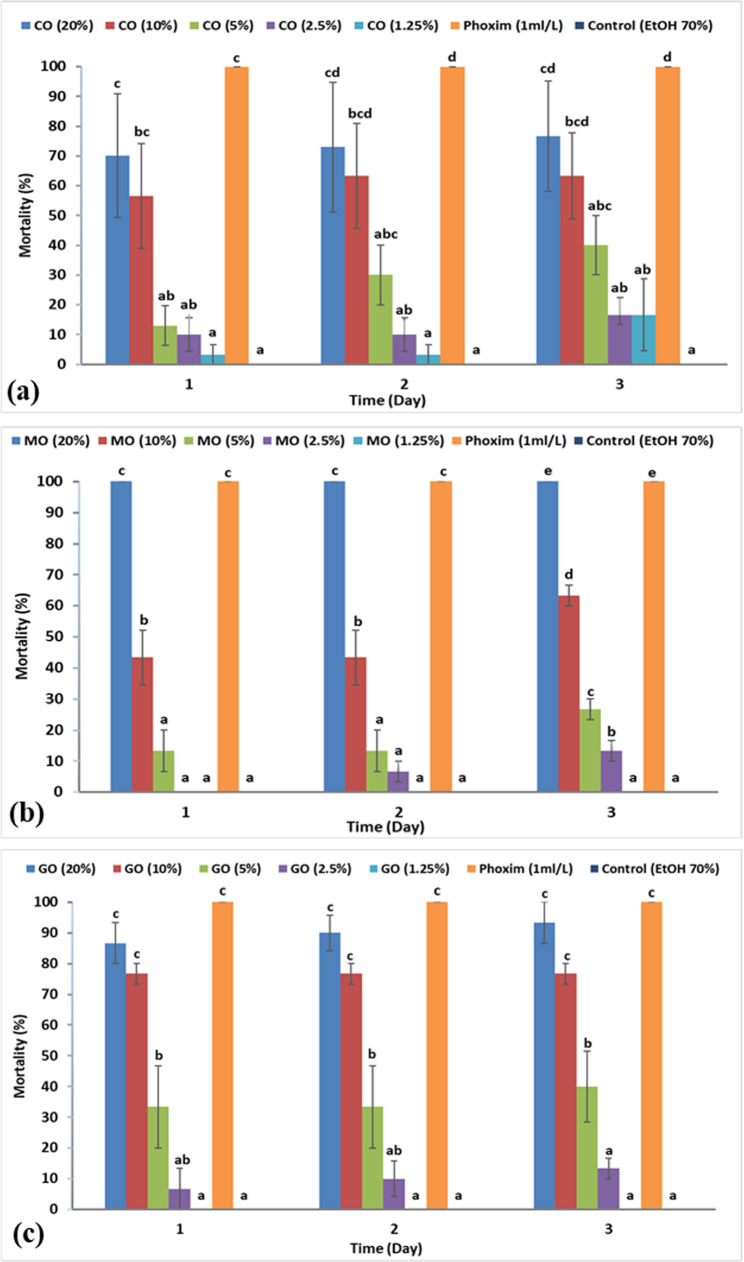
Accumulated mortality percentages of *Rhipicephalus sanguineus* unfed adults exposed to different essential oils for three days: **(a)** clove (CO), **(b)** mint (MO), **(c)** geranium (GO). EtOH 70%: ethyl alcohol

In AIT, the acaricidal efficacy of the three EOs was time and dose-dependent, and the time played a critical role in raising the mortality rate with a significant difference (Fig. 6). The acaricidal efficacy was almost similar in the three tested oils, where the LC_50_ values were 6.93, 6.84, and 6.18% for clove, mint, and geranium EO, respectively, after 3 days (Table 6). The reference acaricide (phoxim 1 ml/L) recorded 100% mortality after 1 day of application; meanwhile, the solvent control (ethyl alcohol 70%) did not record any mortality.

**Table 6:** Lethal concentration of 50% of *Rhipicephalus sanguineus* unfed adults (LC_50_) treated with essential oils, their binary combinations and nanoemulsions after 3, 3 and 7 days, respectively, and synergistic factors of essential oils

Treatment	LC_50_ (%)	Confidence limits (%)	Slope ± SE	SF (Clove)	SF (Mint)	SF (Geranium)
Clove essential oil	6.937	5.82–8.40	1.58 ± 0.15	-	-	-
Mint essential oil	6.847	6.04–7.76	2.77 ± 0.26	-	-	-
Geranium essential oil	6.187	5.43–7.02	2.64 ± 0.25	-	-	-
Clove + Mint (1:1)	6.206	3.89–10.30	2.89 ± 0.21	1.12	1.045	-
Clove + Geranium (1:1)	5.689	2.62–13.71	2.26 ± 0.17	1.21	-	1.08
Mint + Geranium (1:1)	4.933	3.16–7.65	2.12 ± 0.16	-	1.39	1.25
Clove nanoemulsion	1.63	1.4–1.83	3.14 ± 0.28	-	-	-
Mint nanoemulsion	3.92	2.35–6.58	2.61 ± 0.18	-	-	-
Geranium nanoemulsion	4.59	3.93–5.36	2.20 ± 0.25	-	-	-

*SF* Synergistic factor, *SF > 1* Synergism, *SF < 1* antagonism

**Fig. 7:**
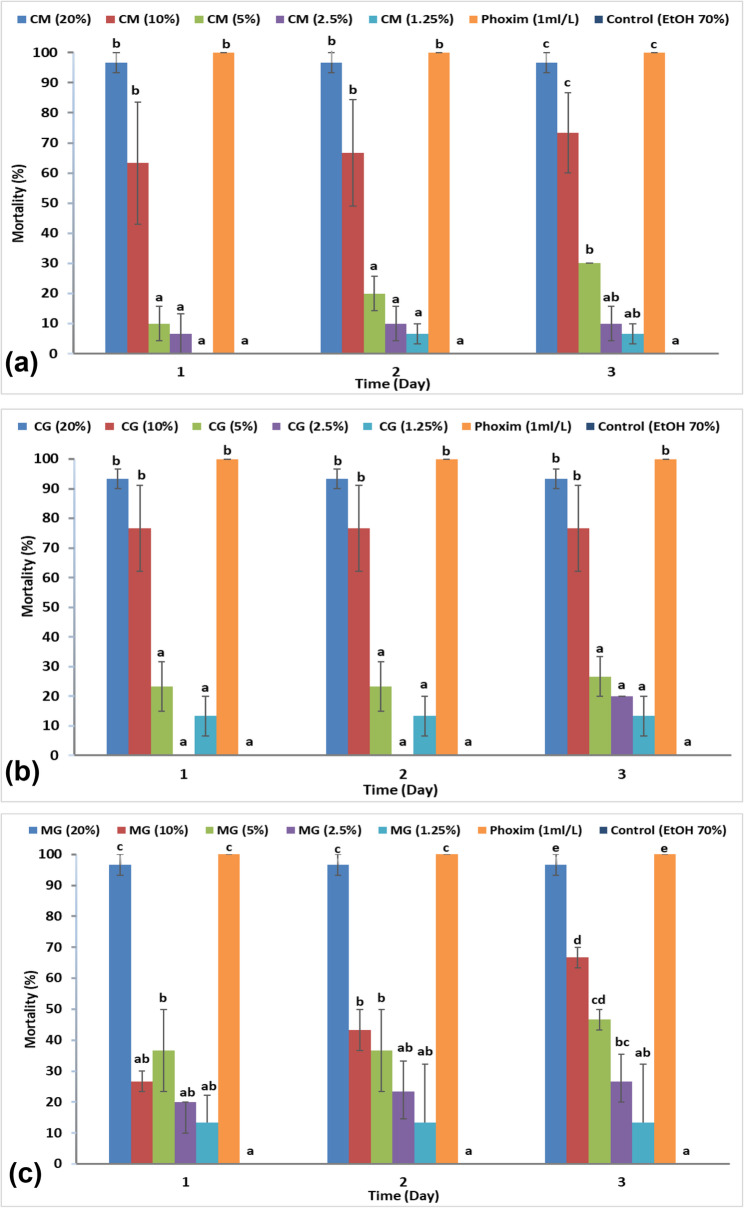
Accumulated mortality percentages of *Rhipicephalus sanguineus* unfed adults exposed to different essential oil binary combinations for three days: **(a)** clove + mint (CM), **(b)** clove + geranium (CG), **(c)** mint + geranium (MG). EtOH 70%: ethyl alcohol

The adulticidal activity of EOs combinations (clove + mint), (clove + geranium), and (mint + geranium) is presented in Fig. 7. The combination of (mint + geranium) showed the highest adulticidal activity with LC_50_ value of 4.9% followed by (clove + geranium) and (clove + mint), where the LC_50_ values were 5.68 and 6.2%, respectively, after 3 days (Table 6). Most of EO combinations showed a synergistic efficacy where the synergistic factor was more than 1. The (mint + geranium) binary combination exhibited the highest synergistic activity with SF (1.39); moreover, the (clove + mint) combinations showed partial synergistic activity against *R. sanguineus* unfed adults with SF (1.04) Table 6.

**Fig. 8:**
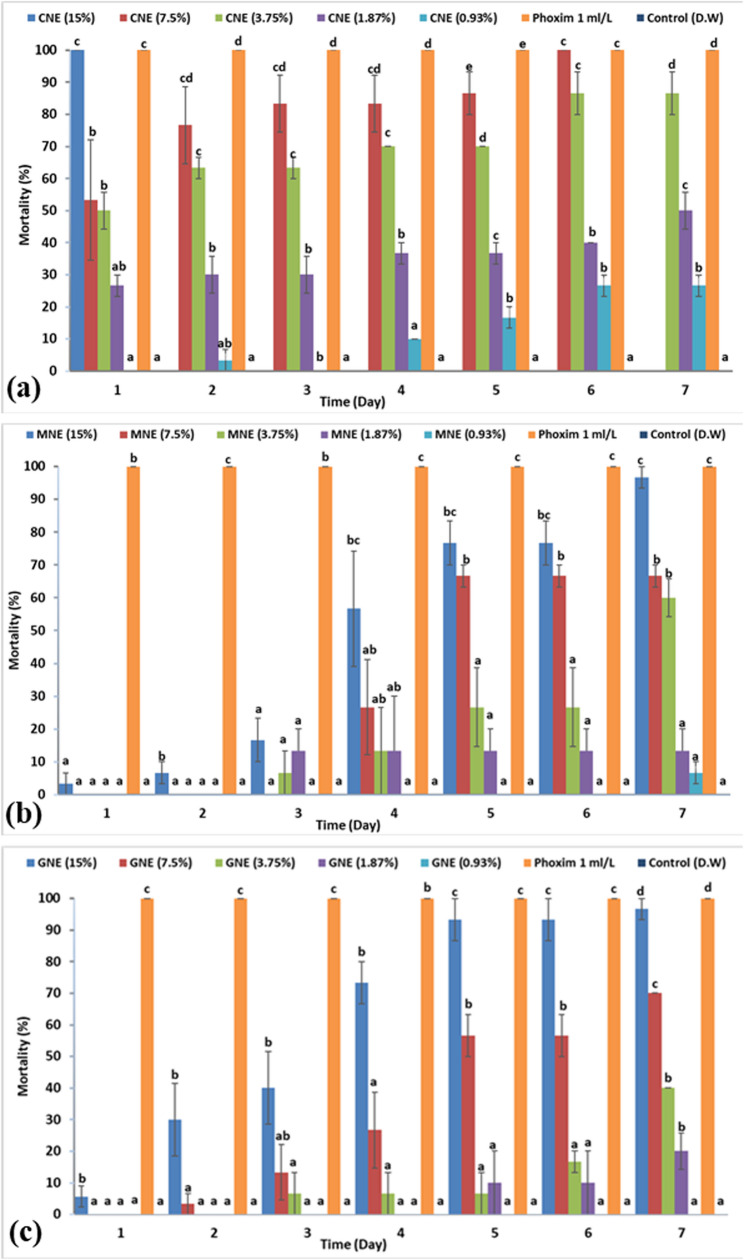
Accumulated mortality percentages of *Rhipicephalus sanguineus* unfed adults exposed to different essential oil nanoemulsions for seven days: (**a**): clove (CNE), (**b**) mint (MNE), (**c**) geranium (GNE). D.W: distilled water

Regarding nanoemulsions, their effect on *R. sanguineus* adults was latent and extended 7 days after application, as shown in Fig. 8, except for clove NE, which recorded its highest mortality (100%) 24 h after its application. The highest adulticidal activity was demonstrated by clove NE, where the LC_50_ value was 1.63% followed by mint NE 3.92%, and then geranium NE, 4.59% after 7 days (Table 6).

According to the LC_50_ results obtained by the in vitro AIT, the most effective materials were clove NE (LC_50_ = 1.63%), followed by (mint + geranium) combination (LC_50_ = 4.933%), and then geranium EO (6.1%).

## Discussion

Chemical acaricides have been widely used for tick control; however, the indiscriminate and extended use has led to several issues such as resistance development and poses risks to non-target organisms [41]. Therefore, various studies are now focused on identifying natural products with acaricidal properties that are safe for the environment. Essential oils from natural products have been investigated for their effectiveness, individually or in combination, as a potential tick control substitute [42]; in addition, they have minimal toxicity to non-target organisms and short residual time in the environment [26]. The present study evaluated clove, mint, and geranium essential oils, along with their binary combinations and nanoemulsion form for their acaricidal activity against *Rhipicephalus sanguineus* larvae and adults.

The GC-MS analysis of clove EO identified eugenol (68.02%) and Caryophyllene (20.19%) as the most abundant constituents. These results are quite similar to Awad et al. [43] and Ikawati et al. [44]where eugenol (50.2%, 89%) and caryophyllene (19.3%, 10%), have the main active components, respectively. It was noted that, from a chemical perspective, eugenol is considered responsible for the acaricidal effect of clove essential oil, as demonstrated by our results and previous studies [45–47]. Eugenol, a phenylpropanoid, has proven insecticidal properties by binding to octopamine receptors, thereby blocking nerve impulses, which results in paralysis of the arthropod [48, 49]. Clove essential oil is effective against larvae and adult ticks because it contains a high concentration (more than 73%) of phenylpropanoids, such as eugenol and eugenol acetate, and certain sesquiterpene hydrocarbon compounds (more than 26%), such as β-caryophyllene, α-humulene, and α-farnesene, which are non-toxic to humans and mammals. This notion was also put forth by Lawal et al. [50] and Aimad et al. [51].

The effect of clove EO against ticks has been investigated; however, efficacy against *R. sanguineus* is lacking. One study by Lambert et al. [52] had evaluated the effect of clove EO against *R. sanguineus* larvae only, with an LC_50_ value of 3.3 mg/mL (0.33%). In the current study, clove EO effect was evaluated on larvae and adults with a higher mortality against larvae where the LC_50_ values were 1.68% for larvae and 6.93% for adults. These results indicate that larvae appear more sensitive to clove oil than the adult stage. Earlier research suggests that the greater impact of oils on larvae compared to adults may return to the fact that adults have a thicker cuticle than larvae, and larvae breathe through their cuticular membrane, which probably permits higher penetration and absorption of EO [53]. This observation aligns with a study by Alimi et al. [13], on *Hyalomma scupense* ticks which reported a mortality of 93.1% for larvae at a concentration of 2.5 mg/mL while 93.7% for adults at 10 mg/mL. Same behavior was observed in *R. microplus*, where clove EO revealed 100% mortality at a concentration of 5 mg/mL for larvae and 50 mg/mL for adults [11]. While clove EO demonstrated rapid acaricidal activity primarily attributed to eugenol, mint EO showed a complementary mechanism involving Pulegone and Menthone. The major active constituents identified in the mint EO of the current study agreed with the same analysis of mint from different geographical locations, including various concentrations [54, 55]. The variation in the chemical constituents and percentages of each compound may relate to physiological and environmental conditions, genetic species, harvesting time, geographical location, and extraction methods [56, 57]. The acaricidal efficacy of mint EO might be attributed to the presence of different active compounds such as Pulegone, l-Menthone, Eucalyptol, β-Pinene, and iso-Menthone, where these major and minor compounds have an essential role in the biological activity of EO [58, 59]. Moreover, the terpenes compounds found in the mint EO influence the octopamine receptor and prevent insect acetylcholinesterase (AChE) from functioning, which leads to paralysis and eventually causes insect death [60]. Mint EO demonstrated good acaricidal activity against larvae and adults with LC_50_ values of 3.52% and 6.84%, respectively. The insecticidal activity of mint against various pests was well documented [61, 62], however, there have been few studies addressing its acaricidal effects on tick larvae only. One study by Koc et al. [16] assessed the impact of mint EO on *Rhipicephalus turanicus* larvae reporting 100% mortality at a concentration of 0.1%. On the other hand, mint EO has an insecticidal effect. Benelli et al. [17] and Govindarajan et al. [63] evaluated its larvicidal activity against *Culex quinquefasciatus*, *Aedes aegypti*, and *Anopheles stephensi*. In another study, mint EO revealed repellent activity against *Culex pipiens* [64]. Likewise, geranium EO showed comparable toxicity levels, implying that the three oils act through related pathways leading to rapid knockdown and mortality. The most abundant components of geranium EO and its concentrations were aligned with the finding of Khalifa et al. [65]. The presence of citronellol and geraniol in the chemical constituents of geranium EO may be responsible for its acaricidal effect [25, 66]. Moreover, it has been shown that geraniol and citronellol are effective plant-based insect repellents [67]. After EO treatment, the volatiles penetrate through the respiratory system and consequently result in breathing difficulty and asphyxiation, leading to death [68]. Geranium EO has shown activity against different tick species [19–21, 69] unlike clove and mint EO, which had fewer studies against ticks. In this study, geranium EO revealed acaricidal activity against *R. sanguineus* ticks with LC_50_ values of 3.91 and 6.18% for larvae and adults, respectively. In a similar study, geranium EO was tested against *R. annulatus* larvae and adults resulting in LC_50_ of 3.43 and 7.53%, respectively [20]. Our results were also consistent with the study of Moawad et al. [69] against *R. annulatus* larvae, where the mortality percentage was 96.33% at 10% concentration. Moreover, geranium EO affects the reproductive efficiency of *R. microplus* ticks [19].

Each essential oil possesses a unique chemical composition and distinct mechanism of action, which may target different physiological pathways in arthropods. A binary combination of two or more compounds can create novel botanical pesticides containing substances that have synergistic effects. Different studies have demonstrated that mixtures of substances of plant origin have synergistic actions against a wide range of target organisms, including bacteria [70], fungi [71], nematodes [72] and insects [22, 73]. In this study, the effect of EO combinations at a ratio of 1:1 was evaluated against *R. sanguineus* larvae and adults to determine the improvement of their efficacy. The primary goal of employing synergistic combinations is to utilize a low concentration of the tested materials, reducing costs and increasing efficacy against the target organisms. Additionally, the greater chemical complexity of mixtures lowers the chances of resistance development [74]. Interestingly, here all EO combinations exhibited larvicidal and adulticidal activity against *R. sanguineus* ticks, with lower LC_50_ values being achieved and synergistic factors exceeding one. Among the combinations tested against *R. sanguineus* larvae was (clove + mint), which demonstrated the highest acaricidal activity which may be attributed to the complementary actions of their major constituents (eugenol, the dominant compound in clove acts on octopamine receptors [48, 49], and pulegone and menthone, the principal components of mint oil, act on acetylcholinesterase (AChE) [60]) which results in paralysis and death. Moreover, the combination of (mint + geranium) exhibited the highest adulticidal activity against *R. sanguineus* adults. The enhanced acaricidal activity of the (geranium + mint) binary combination likely results from the multi-target actions of their major constituents (citronellol and geraniol in geranium oil, and pulegone and menthone in mint oil) which act on both neural and metabolic systems. These interactions may improve cuticular penetration and volatility, leading to faster knockdown and higher mortality. In addition, both mint and geranium are rich in oxygenated monoterpenes, complementary interaction between oxygenated monoterpenes of different chemical classes producing greater efficacy than either oil alone. Similar synergistic enhancements have been reported in mixtures of essential oils rich in monoterpenes and monoterpenoids against various arthropod pests [75].

To our knowledge, this study is the first to evaluate the synergism between clove, mint and geranium EO combinations against *R. sanguineus* ticks. Several authors discovered that synergetic interaction between the various oil ingredients, such as terpenes and phenylpropanoids, greatly increased their acaricidal effectiveness when they were combined [12, 76, 77]. Many studies demonstrated the acaricidal activity and synergism of EO combinations against *R. sanguineus* ticks. The effect of (geranium + oregano + thymol) combination was conducted by Gadelhaq et al. [25] against eggs, larvae, and adults of *R. sanguineus* ticks with LC_50_ values of 2.81, 2.44, and 13.4 mg/ml, respectively. This was consistent with the results of our study, where the combination effect was higher in larvae than in adults. Other EO combinations proved their synergistic activity against different tick species, such as the geranium and sesame oil combination against *R. annulatus* ticks [20], the carvacrol and thymol mixture against *Amblyomma sculptum* and *Dermacentor nitens* [78], and lemon grass and clove EOs against *R. microplus* adults [49]. Although clove, mint, and geranium EOs exhibited good acaricidal activity, they have some limitations for their use, such as water insolubility, active ingredient breakdown, and short longevity [79]. To resolve these issues and enhance the acaricidal efficacy of EOs, they were used in nanoemulsion form. It is well recognized that developing formulations using nanotechnology improves the effect of EOs and allows the use of low concentrations, raising the possibility of economic biopesticides [80]. Nanoemulsions of clove, mint and geranium were characterized by TEM and particle size distribution to prove the EOs had attained the nanostructure. The average particle size from TEM images ranges from 47.8 to 173 nm, which indicates successful preparation of nanomaterials and follows the fact that the excellent NE droplet size ranges from 20 to 200 nm [81]. The size of the particles affects the absorption, biocompatibility, and bioactivity of the loaded drug. It has been demonstrated that the smaller the size of the prepared NEs, the higher the chance of being in contact with the surface, which improves their absorption and promotes mobility with a gradual release, ultimately increasing systemic activity [82, 83]. The particle size of NE ranges from 10 to 500 nm according to previous studies [84]. The particle size and polydispersity index (PDI) were (80 nm & 0.286), (213.2 nm &0.331), and (244.2 nm & 0.55) for clove, mint, and geranium NEs, respectively. According to low PDI values for all NEs indicate the overall stability and homogeneity of the nanoformulation as the PDI value ≤ 0.7, and hence the efficiency of the particles to penetrate the cell membrane will be more effective due to the reduction of the particle size.

Herein, the clove, mint, and geranium NEs revealed acaricidal activity against *R. sanguineus* larvae and adults higher than the ordinary oils. The effect of NEs on larvae was recorded 24 h after their application. In contrast, the effect against adults was latent and extended 7 days after their application, except for clove NE where the adult mortality was recorded after 24 h. The rapid effect of clove NE versus the delayed effect of mint and geranium NE may be firstly returned to clove NE rich in eugenol which may employ neurotoxic effect that leads to rapid paralysis and death, while mint and geranium NEs affect physiological and metabolic route (interference with metabolism, feeding inhibition, or reproductive disruption). Secondly, clove NE showed the smallest droplet size and the lowest PDI compared with mint and geranium NE which allowed faster penetration and early toxicity.

The latent effect of NE was observed in the study conducted by Abdel-Ghany et al. [28] evaluating the impact of myrrh, patchouli, and cypress NEs against unfed adults of *R. sanguineus* where the effect began to appear 72 h after treatment, reporting LC_50_ values of 4.17, 8.57, and 5.04% after 7 days, respectively. Although the efficacy of mint and geranium NEs against adults was late and showed their highest mortality after 7 days post-treatment, they have the benefit of using concentrations less than those used in ordinary oil, and this seems better from an economic point of view. The authors hypothesized that the extended effect observed for mint and geranium NEs may be indicative of improved stability and a sustained release of compounds compared to the pure oils, though this requires further investigation through long-term stability studies.

To our knowledge, no data about the effect of clove and mint NE against ticks has been noted, and only a few studies have focused on geranium NE. In this study, the effect of clove NE was higher than that of ordinary oil, and the larvae were more sensitive to clove NE than the adults, where the LC_50_ values were 0.79 and 1.63%, respectively. This was in the same line as Wahba et al. [85] who reported that clove NE was more effective than that of ordinary oil against *Spodoptera littoralis* (Boisd) (Lepidoptera: Noctuidae), with LC_50_ values of 1.77 and 2.18%, respectively. Furthermore, the research conducted by Hassan et al. [86] reported that clove NE exhibits higher insecticidal activity against the fleas, *Xenopsylla cheopis*, than clove oil, with LC_50_ value of 36.26 and 26.42 µg/ml for oil and NE, respectively. Doungnapa et al. [87] also emphasized that clove NE was more effective than EO against African red mite *Eutetranychus africanus*, where the same concentration of 2% resulted in 100, and 71.7% mortality, respectively.

For mint NE, it also produced a higher acaricidal effect against larvae than adults with LC_50_ values of 0.363 and 3.92%, respectively. Mint NE has been studied as an effective and eco-friendly material against mosquito larvae [88–90]. Furthermore, the research conducted by Mohafrash et al. [91] demonstrated the effectiveness of mint NE against *Culex pipiens* and *Musca domestica* larvae, with LC_90_ values of 91.12 µg/mL and 148.74 µg/mL, respectively. According to Louni et al. [92], mint NE was more toxic for *Ephestia kuehniella* (Lepidoptera: Pyralidae) than ordinary oil, and the LC_50_ value was 14,068 ppm for mint NE and 21,352 ppm for mint oil.

Regarding geranium NE, it exhibited relatively low toxicity against larvae and adults compared with clove and mint NE. The geranium NE was more toxic for larvae than adults and the recorded LC_50_ values were 3.17% and 4.5%, respectively. These results agree with the study of Ibrahium et al. [20] who tested the effect of geranium NE against *R. annulatus* ticks where the larvae were more sensitive than adults with LC_50_ values of 1.68% and 5.60%, respectively. Geranium NE exhibited LC_50_ of 1.5% against house fly larvae, and 0.19% for *Culex pipiens* larvae [93]. Moreover, the geranium loaded on solid lipid nanoparticles (SLN) was tested against the potato tuber larvae, *Phthorimaea operculella*, the SLN was more effective than geranium alone [94]. The differences among the three NEs in terms of droplet size distribution and PDI may explain the variation in their biological activity. For instance, NEs with smaller droplets and higher stability likely provide more uniform and sustained release of active ingredients, resulting in stronger acaricidal effects. The superior efficacy of clove NE correlates directly with its optimal nanoscale properties: the smallest droplet size (80 nm) and the lowest PDI (0.286), which would promote better cuticular penetration and stability. In contrast, the relatively lower efficacy of geranium NE may be attributed to its larger droplet size (244.2 nm) and higher PDI (0.55), indicating a less stable and less homogenous formulation. Finally, these observations highlight how differences in chemical composition and formulation properties influence the biological performance of essential oils and their nanoemulsions against *R. sanguineus*.

## Conclusion

This study evaluated the acaricidal effects of clove, mint, and geranium essential oils (EOs), along with their binary combinations and nanoemulsion forms, against *Rhipicephalus sanguineus* larvae and unfed adults. According to LC_50_ values, the most effective materials against larvae were mint NE (LC_50_ = 0.36%), followed by (clove + mint) EO combination (LC_50_ = 1.43%) and then the clove EO (LC_50_ = 1.68%). While the most effective materials against adults were clove NE (LC_50_ = 1.63%), followed by (mint + geranium) combination (LC_50_ = 4.933%), and then geranium EO (6.1%). Therefore, these essential oils, particularly clove NE, represent promising, eco-friendly alternatives to synthetic acaricides for the integrated management of *R. sanguineus.* Future work will focus on developing combinations of nanoemulsions and critically evaluating their safety and in vivo efficacy to facilitate practical application.

## Data Availability

Data will be made available on request.
